# Lockdown measures and air quality: evidence from Italian provinces

**DOI:** 10.1007/s12076-021-00267-4

**Published:** 2021-02-21

**Authors:** Maurizio Malpede, Marco Percoco

**Affiliations:** 1 GREEN, Università Bocconi, Milano, Italy; 2 Department of Social and Political Science and GREEN, Università Bocconi, Milano, Italy

**Keywords:** COVID-19, Air quality, Particulate matter, Pandemics, Pollution, I18, Q53

## Abstract

The aim of this short communication is to estimate the effects of the implementation of more restrictive lockdown measures on pollution levels in Italy. Using a time series of weekly concentrations of PM_10_, PM_2.5_ and NO_2_ for the period 2016–2020 across 71 provinces, we find that the introduction of lockdown measures reduced the air concentration levels of PM_10_ and NO_2_ by 17–18%, while their effect on PM_2.5_ remains unclear. These results indicate that the lockdown had a significant positive impact in terms of lives saved and improved air quality.

## Introduction

Few cases in human history have generated such a pervasive slowdown in consumption and production as the COVID-19 pandemic. Analysis of the temporary and persistent effects of sizeable health shocks on local socio-economic systems in terms of the shape of the development patterns and the capacity to absorb such shocks has generated a large body of literature.^1^ However, the environmental consequences of pandemics have not been extensively investigated (Helm 2020).

At the time of writing (28 December 2020), the number of cases of COVID-19 reported worldwide exceeded 80 million, and more than 1.7 million deaths have been registered.^2^ The speed of the geographical spread of the disease is unprecedented; the first case was reported on 8 December 2019 in Wuhan, and on 28 January 2020, more than 800 cases were reported internationally in Japan, South Korea, the USA, Taiwan, Hong Kong, Macau, Singapore and Vietnam, although the majority of cases were concentrated in China. In an attempt to contain the spread of the virus, residents of Wuhan were placed under quarantine from 23 January. This was immediately followed by quarantine measures in the cities of Huanggang and Ezhou and the cancellation of Chinese New Year celebrations.

This deadly pandemic has happened at a time when the concentration of pollution in urban areas has been steadily increasing over the past decades, imposing a severe death toll in all major cities in both developed and developing countries.^3^ A number of newspaper articles have been published recently on the observed reduction in the air concentrations of NO_2_ and PM_2.5_ due to the widespread adoption of lockdown measures and semi-voluntary social distancing, which have reduced mobility and overall levels of economic and human activity.^4^ Thus, the introduction of lockdown measures may have potentially saved a significant number of lives due to both the containment of COVID-19 and a reduction in pollution-induced deaths (Bosetti et al. 2020).

Although recent studies have focused on the effects of complete lockdowns on air pollutant levels in various areas in China and Europe (Bosetti et al. 2020), this paper is the first to provide empirical estimates on the impact of *different degrees* of anti-COVID-19 measures in Italy on pollution levels, measured by concentrations of PM_10_, PM_2.5_ and NO_2_. This is achieved using a comprehensive dataset containing the pollution levels across all Italian provinces recorded at weekly intervals between 2016 and early 2020. We match the weekly pollution data for each Italian province with the dates on which measures to restrict the spread of COVID-19 were introduced. The different dates on which each Italian province and region adopted specific measures are reported on the official Italian government website.^5^

We found a significant contraction of 17–18% of air concentration levels of PM_10_ and NO_2_ compared to the concentrations measured before the outbreak of COVID-19.

## Methodology and data

In this paper, we aim to identify the impact of lockdown measures on the air concentrations of three pollutants in Italian provinces. To investigate these relationships, we estimated several versions of the following baseline equations:

1 $$Pollutant_{p,t} = \alpha + \beta Treatment_{pt} + \delta_{t} + \gamma_{p} + {\text{f}}\left( {{\text{trend}}} \right) + \varepsilon_{p,t}$$

2 $$Pollutant_{p,t} = \alpha + \beta Measure_{pt} + \delta_{t} + \gamma_{p} + {\text{f}}\left( {{\text{trend}}} \right) + \varepsilon_{p,t}$$

where the $Pollutant_{p,t}$ represents the average weekly air concentration at province level expressed in terms of mg/m^3^ of one of the following pollutants: PM_10_, PM_2.5_ and NO_2_. Our treatment in Eq. (1) is represented by the indicator variable $Treatment_{p,t}$, which takes value equal to 1 after the introduction of shelter-in-place policy (i.e. the complete halt of all non-essential production) in Italian province p and value equal to 0 before the implementation of the lockdown.

Our treatment in in Eq. (2) is represented by the categorical variable *Measure*_*p,t*_, which shows the degree of restrictions imposed by measures implemented in province *p* at time *t*. It takes the value 0 before the outbreak of the disease and a value between 1 and 5 after the implementation of lockdown measures. The degrees of restrictions imposed by the measures take the following incremental values: (1) social distancing is encouraged with no compulsory restrictions; (2) public events are banned; (3) schools and theatres are closed; (4) lockdown is ordered; (5) further restrictions on all non-essential production.

### Measure of the severity of the restrictions

In this paper, we consider five levels of lockdown intensity, ranging from suggested social distancing, which was first encouraged for people living in Lombardy and Veneto, to the complete halt of all non-essential production. The definition of the “Measure” variable relies on the timeline of the restrictions occurred in Italy from February 24th until May 4th described in the “Appendix”. Specifically, the variable $Measure_{p,t}$ takes a value of 1 when social distancing was encouraged (no compulsory restrictions). On 21 February, public events were banned and schools closed in 10 cities within the provinces of Lodi and Piacenza in Lombardy. In our regression, the variable *Measure* takes a value of 3 for these provinces and a value of 1 for other provinces.^6^ On the following day, these two provinces and one province in the Veneto region were pronounced red zones and a complete lockdown was implemented. Public events were also banned in all provinces in Lombardy. In our regression, the variable *Measure* takes a value of 4 for Lodi and Piacenza starting from 23 February and a value of 3 for all other provinces in Lombardy. On 25 February, public events (including sport) were banned and schools and universities closed in five Italian regions, namely Emilia-Romagna, Friuli-Venezia Giulia, Veneto, Piemonte and Liguria.^7^ In our regression, the variable *Measure* takes a value of 4 for the provinces of those regions too. On 1 March, the ban on all public events was extended to the entire Italian territory.^8^ On 4 March, the closure of public schools was also extended to all Italian regions. Our variable *Measure*, therefore, takes a value of 4 for all Italian provinces starting from 4 March. On 5 March, a shelter-in-place order was issued for the region of Lombardy and 14 provinces in the north of Italy, namely Modena, Parma, Piacenza, Reggio nell’Emilia, Rimini, Pesaro e Urbino, Alessandria, Asti, Novara, Verbano-Cusio-Ossola, Vercelli, Padova, Treviso and Venezia. In our regression, the variable *Measure* takes a value of 5 for those provinces. Finally, the complete lockdown was extended to all Italian regions on 11 March.^9^

The dummy variables $\delta_{t} {\text{ and }}\gamma_{p}$ indicate full sets of week-of-the-year- and province-specific fixed effects. These variables are of paramount importance because they capture week-specific regularities and time-invariant characteristics at the province level. $\varepsilon_{t,p}$ is an IID error term, such that, using the panel least squares estimator, we retrieve an estimate for parameter *β* that measures the impact of lockdown measures and intensity on pollution concentrations. It should be noted that in several specifications of Eq. (1), we include province-specific quadratic time trends—the function *f(trend)* in Eq. (1)—to account for possible local temporal trends in the concentration of pollutants.

Equation (1) is estimated using data from the European Environmental Agency (EEA) on 71 provinces for the period 2014–2020. Table 1 presents the descriptive statistics for the main outcome variables, which are the air concentrations of PM_10_, PM_2.5_ and NO_2_. The concentrations of PM_10_ and PM_2.5_ were slightly reduced, and a more substantial reduction in NO_2_ concentrations was observed.

**Table 1:** Summary statistics

Variable	Mean		Min	Max	Std	Obs
	Pre Lockdown	Post Lockdown				
PM_10_	28.81	27.52	4	164.16	19.91	2262
PM_2.5_	16.27	15.74	0	74.5	9.34	915
NO_2_	29.82	19.19	2.57	99.67	13.48	3220

### Testing for existing trends

In this section we test for the common trend assumption of the dependent variables (i.e. levels of air pollutants in the air) for each of the 71 Italian provinces. The common trend test is performed through the estimation of a panel regression including leads and lags.

**Fig. 1:**
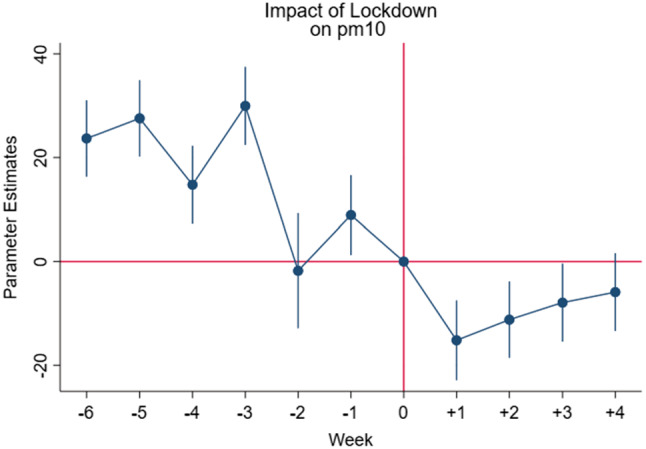
Effects of the introduction of the restrictive measures on the concentration levels of PM_10_

**Fig. 2:**
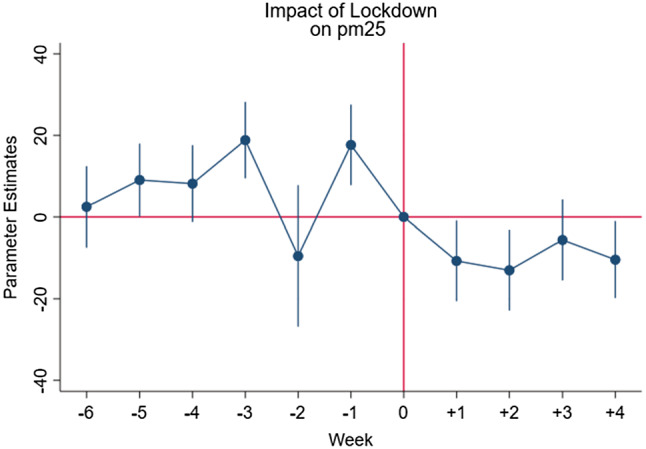
Effects of the introduction of the restrictive measures on the concentration levels of PM_2.5_

**Fig. 3:**
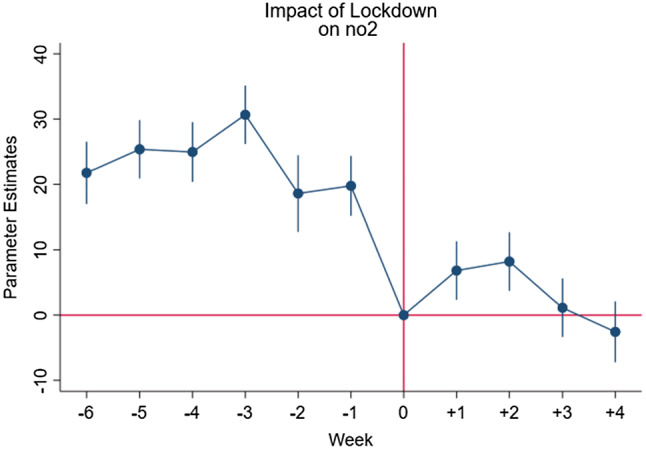
Effects of the introduction of the restrictive measures on the concentration levels on NO_2_

Hence the following equation is estimated:

$$Pollutant_{p,t} = \alpha + \mathop \sum \limits_{k = t - 5}^{t + 2} \beta_{k} D_{p,k} + \delta_{t} + \gamma_{p} + {\text{f}}\left( {{\text{trend}}} \right) + \varepsilon_{p,t }$$

where $D_{p,k}$ are dummy variables which correspond to each of the moths before and after the policy is introduced; Specifically, k takes value equal to 0 in the last week prior to the introduction of the policy for each specific Italian province (i.e. 15–22 February 2020) and it ranges from -5 (5 weeks prior to lockdown) to + 2. The $\beta_{k}$ coefficients explain the average pollution levels in the weeks prior to the lockdown and after the latter has taken effect. This fixed effect estimation allows us to check for the existence of a preexisting time trend *before* the intervention of the Italian government to limit the spread of covid-19. Figs. 1, 2 and 3 show graphically the eventual existence of a common trend.

## Results

We start our analysis with a version of Eq. (1) where the treatment variable, $Treatment_{pt}$, is binary and takes the value 0 before the lockdown and 1 afterwards. Table 2 shows the results of three equations differing only in terms of the dependent variable. Our results show that lockdown measures had an impact on the concentrations of PM_10_ and NO_2_ of an order of magnitude of − 5.125 and − 5.375 μg/m^3^, respectively. However, the impact on PM_2.5_ is not statistically significant, with usual thresholds on the *p*-value of the coefficient of interest.

**Table 2:** The impact of lockdown on pollutant concentration

	(1)	(2)	(3)
	PM_10_	PM_2.5_	NO_2_
	Coef./SE	Coef./SE	Coef./SE
Lockdown	− 5.125*** (1.749)	− 1.996 (1.602)	− 5.375*** (1.193)
Date FE	Yes	Yes	Yes
Province Month trend	Yes	Yes	Yes
Observations	2262	915	3220

This table shows the effects of lockdown on air pollutant concentration in Italy at province level. Significant at ***p < 0.01, **p < 0.05, *p < 0.1

Table 3 presents the parameter estimates for Eq. (2) in which the treatment variable is intended to capture differential intensity in the degrees of lockdown. The estimated coefficients for PM_10_ and NO_2_ have magnitudes slightly below those reported in Table 2 as a province that implemented a measure of degree 4, which was the case for the vast majority of provinces, experienced a reduction of − 3.424 μg/m^3^ in the concentration of PM_10_ and − 4.392 μg/m^3^ in the concentration of NO_2_. Furthermore, in this case, the coefficient indicating the impact of lockdown restrictions on the concentration of PM_2.5_ is estimated at − 0.588, which is significant at a 5% significance level.

**Table 3:** The impact of lockdown intensity on pollutant concentration

Table: impact of restrictive anti-COVID-19 measures on air quality in Italy			
	(1)	(2)	(3)
	PM_10_	PM_2.5_	NO_2_
	Coef./SE	Coef./SE	Coef./SE
Lockdown measure	− 0.856*** (0.307)	− 0.588** (0.261)	− 1.098*** (0.222)
Date FE	Yes	Yes	Yes
Province Month trend	Yes	Yes	Yes
Observations	2262	915	3220

This table shows the effects of restrictive measures on air pollutant concentration in Italy at province level. Significant at ***p < 0.01, **p < 0.05, *p < 0.1

One explanation of the differential effects that the restrictive measures had on the concentration of PM10 and PM2.5, respectively lies on the different sources of the two pollutants.

While PM10 levels are greatly associated with the use of cars and other oil-based transport means, which have been consistently limited since February 24th; PM2.5 levels are principally due to agricultural and industrial production processes along with the combustion of coal and natural gas. Restrictions on the economic sectors did only occur gradually and partially, as described in the “Appendix”. As a result, provinces with great industrial concentration showed a lower reduction in PM2.5 compared to areas with a minor presence of industries.

We interpret the coefficients in Tables 2 and 3 in light of the descriptive statistics in Table 1. For simplicity, considering only the coefficients in Table 2, we can conclude that the introduction of lockdown measures reduced PM_10_ levels by 17.79% and NO_2_ levels by 18.02% compared to pre-lockdown concentrations.

Figures 1, 2 and 3 show the weekly parameter estimates of the effects of the introduction of the restrictive measures on the concentration levels of PM_10_ PM_2.5_ and NO_2,_ respectively. Taken together the three figures highlight that the negative effects of the restriction on air pollutant concentration occurred during the first 2 weeks, while slowly decreasing over the other 2 weeks. These results, once again, confirm the effectiveness of the lockdown measures implemented in Italy in terms of improvements in air quality levels.

## Conclusions

The COVID-19 pandemic is posing a fatal toll and will likely have substantial negative repercussions for local and national economies (Kristalieva, 2020). Although this large-scale experiment does not allow us to distinguish between the effect of a reduction in social contact (Anderson et al. 2020; The Lancet Editorial Board 2020) and that of the contraction of production and consumption in Italy, our results point to a positive and substantial role of voluntary and imposed social distancing not only in terms of containing the spread of COVID-19 but also in terms of improving air quality and a possible subsequent health effect.

According to a recent report by the EEA (2019), fine particulate matter (PM_2.5_ and PM_10_) alone caused approximately 374,000 of deaths in the European Union (EU) in 2016. However, the general reduction in fine particulate matter observed between 2015 and 2016 reduced the number of premature deaths in the EU by approximately 17,000 (a 4.5% absolute reduction). The EEA also highlights that fine particulate matter concentrations were 67% higher in the Italian regions of Emilia-Romagna, Piemonte and Lombardy compared with EU limit values. Thus, a reduction in pollutant concentration would highly benefit those regions.

However, it is important to stress that the reductions in air pollutants observed for Italy in 2020 are likely to be short-lived as they do not reflect long-term structural changes in the economy.^10^ While it remains to be seen how long social distancing behaviours will be practised, these behavioural changes alone will not be enough to drive emissions down and improve air quality.

## Appendix

### Timeline of covid-19 related restrictions in Italy and definition of the associated “Measure” variable

The COVID-19 outbreak in Italy was first detected in the province of Lodi, prompting national and local authorities to implement a series of lockdown measures spanning the months of February and March.^11^ Italy first issued a region-specific lockdown on 8 March in the region of Lombardy (with 16 million residents) and 14 additional provinces in the regions of Emilia-Romagna and Veneto. This was soon followed by a nationwide lockdown on 11 March. Travel within Italy was banned except for health reasons or urgent matters.

Schools and universities were closed in Lombardy, Veneto and Emilia-Romagna on 23 February and in Bologna, the major southern cities of Palermo, Napoli and Bari and the region of Abruzzo on 27 February. Starting from 4 March, all schools and universities across the Italian territory were closed. From 21 March, the Italian government further tightened anti-COVID-19 measures, imposing a closure of non-essential businesses; only supermarkets, banks, pharmacies and post offices were allowed to remain open. This last measure was introduced earlier, on 15 March, in the region of Campania.

Until 3 May, people in Italy were only permitted to leave their homes under certain circumstances, including solitary exercise close to home (within 200 m), grocery shopping or visits to the doctor. By law, all residents on the Italian territory had to print a certificate declaring their reason for leaving the house, which would be checked by police. Those who violated the lockdown were subject to a fine of between €400 and €3000 or, in the worst case, three months imprisonment. From 14 April, the Italian government began to relax restrictions, allowing bookshops, stores selling clothing for children and infants and other small shops to reopen. The forestry industry was allowed to resume production.

The main steps leading to the complete lockdown and the temporary halt of non-essential production can be summarised as follows^12^:

- 22 February: Lodi and Piacenza provinces declared red zones.
- 23 February: Public events banned in the regions of Lombardy, Veneto and Emilia-Romagna.
- 27 February: Schools closed in Palermo, Napoli, Bari, Bologna and Abruzzo.
- 04 March: Schools closed nationwide.
- 08 March: Complete lockdown in Lombardy and 14 provinces in the north of Italy.
- 11 March: Complete lockdown extended nationwide.
- 15 March: Further restrictions in two provinces in Campania (Avellino and Salerno).
- 21 March: Halt of all non-essential production.

## Footnotes

1 Examples of studies that consider local long-term effects of plague from a macroeconomic perspective include Alfani (2013) and Alfani and Percoco (2019). On a microeconomic scale, Percoco (2017) tested the hypothesis that in-utero exposure to health shocks decreases individuals’ cognitive capabilities by analysing the persistent effect of the Spanish flu on the capacity to accumulate. Almond et al. (2018) provide an interesting review of the literature on such topics.

2 The official number of coronavirus cases, deaths and recovered people are retrieved from: https://www.worldometers.info/coronavirus/?

3 See, e.g. Dominici et al. (2014) and World Health Organization (2016).

4 NASA, Airborne Nitrogen Dioxide Plummets Over China, Retrieved 5 March 2020 from https://earthobservatory.nasa.gov/images/146362/airborne-nitrogen-dioxide-plummets-over-china; see also Copernicus Atmosphere Monitoring Service, Amid Coronavirus Outbreak: Copernicus Monitors Reduction of Particulate Matter (PM_2.5_) over China, Retrieved 5 March 5 2020 from https://atmosphere.copernicus.eu/amid-coronavirus-outbreak-copernicus-monitors-reduction-particulate-matter-pm25-over-china

5 Timeline of the introduction of specific anti-COVID-19 measures by the Italian government. http://www.governo.it/it/coronavirus-misure-del-governo

6 Available at http://www.quotidianosanita.it/scienza-e-farmaci/articolo.php?articolo_id=81547

7 Available at https://www.ansa.it/sito/notizie/sport/2020/02/25/coronavirus-sport-a-porte-chiuse-in-cinque-regioni_3328c023-1f8d-4a3a-a377-ed9c0717b498.html

8 Available at http://www.quotidianosanita.it/scienza-e-farmaci/articolo.php?articolo_id=81762

9 Available at http://www.governo.it/it/articolo/coronavirus-firmato-il-dpcm-8-marzo-2020/14266 and http://www.quotidianosanita.it/scienza-e-farmaci/articolo.php?approfondimento_id=14527

10 On June 3, all bans were lifted. However, social distancing behaviours among citizens continue to be encouraged. Moreover, all production activities have resumed.
